# Optimal search strategies for identifying sound clinical prediction studies in EMBASE

**DOI:** 10.1186/1472-6947-5-11

**Published:** 2005-04-29

**Authors:** Jennifer L Holland, Nancy L Wilczynski, R Brian Haynes, The Hedges Team

**Affiliations:** 1 Health Information Research Unit, Department of Clinical Epidemiology and Biostatistics, Faculty of Health Sciences, McMaster University, Hamilton, Ontario, L8N 3Z5 Canada; 2 Department of Medicine, Faculty of Health Sciences, McMaster University, Hamilton, Ontario, L8N 3Z5 Canada

## Abstract

**Background:**

Clinical prediction guides assist clinicians by pointing to specific elements of the patient's clinical presentation that should be considered when forming a diagnosis, prognosis or judgment regarding treatment outcome. The numbers of validated clinical prediction guides are growing in the medical literature, but their retrieval from large biomedical databases remains problematic and this presents a barrier to their uptake in medical practice. We undertook the systematic development of search strategies ("hedges") for retrieval of empirically tested clinical prediction guides from EMBASE.

**Methods:**

An analytic survey was conducted, testing the retrieval performance of search strategies run in EMBASE against the gold standard of hand searching, using a sample of all 27,769 articles identified in 55 journals for the 2000 publishing year. All articles were categorized as original studies, review articles, general papers, or case reports. The original and review articles were then tagged as 'pass' or 'fail' for methodologic rigor in the areas of clinical prediction guides and other clinical topics. Search terms that depicted clinical prediction guides were selected from a pool of index terms and text words gathered in house and through request to clinicians, librarians and professional searchers. A total of 36,232 search strategies composed of single and multiple term phrases were trialed for retrieval of clinical prediction studies. The sensitivity, specificity, precision, and accuracy of search strategies were calculated to identify which were the best.

**Results:**

163 clinical prediction studies were identified, of which 69 (42.3%) passed criteria for scientific merit. A 3-term strategy optimized sensitivity at 91.3% and specificity at 90.2%. Higher sensitivity (97.1%) was reached with a different 3-term strategy, but with a 16% drop in specificity. The best measure of specificity (98.8%) was found in a 2-term strategy, but with a considerable fall in sensitivity to 60.9%. All single term strategies performed less well than 2- and 3-term strategies.

**Conclusion:**

The retrieval of sound clinical prediction studies from EMBASE is supported by several search strategies.

## Background

Clinical prediction guides (CPGs), also known as clinical prediction rules or clinical decision rules, are increasingly sought by frontline clinicians to assist in their decision making process. They provide an objective standard by which to gauge which elements in a patient's history, physical examination and laboratory tests are the most important in forming an accurate clinical assessment [1]. CPGs are created by way of deriving the rule, testing or validating the rule, and assessing the impact of the rule on clinical behaviour (impact analysis) [2-5]. CPGs vary in complexity, but those that require simple calculations on the part of the user are most recommended by CPG advocates [5,6].

CPGs can serve as decision aids for determination of causation, diagnosis, prognosis, or patient responsiveness to treatment [1-3]. Some CPGs have been tailored for online or personal digital assistant (PDA) tools to aid in bedside decision-making [6]. Currently available CPGs cover a wide range of topics. For example, guides help to establish the pretest probability of pulmonary embolus [7], to determine the treatment for pharyngitis [8] and to rule out the need for unnecessary radiography for knee injuries ("Ottawa Knee Rule") [9]. CPG advocates state that, when rigorously created and appropriately applied, CPGs have the potential to influence clinical opinion, change clinical behaviour and increase efficiency while preserving or improving quality patient care and satisfaction [5].

Retrieving CPG studies from the medical literature is problematic for several reasons. First they are relatively few in quantity in comparison to other types of studies and reports posted in major, online, clinical literature databases. For example, EMBASE contains more than 9 million records and is up dated by 6000 – 8000 records per week, spread over more than 4600 journal titles [10]. Second, only a fraction of CPG studies are of high quality [1,3-5,11]. A third problem interfering with retrieval is the plurality in terminology associated with CPG studies including test, rule, index, equation, scale, score, profile, prognosis, risk estimate, and model. Fourth, and coupled with varied terminology, is the lack of standardized controlled indexing vocabulary assigned to CPG studies, which precludes their easy extraction from large databases. Finally, studies show that clinicians lack searching skills and have little time to devote to the task of finding high quality studies on which to base their clinical practice [12,13].

Several research groups have identified search strategies ("hedges", in library parlance) for MEDLINE for topics such as etiology [14], diagnosis [15], prognosis [16,17], treatment [18] and review articles [19]. Our own studies of MEDLINE retrieval [16,17] led to the creation of the PUBMED Clinical Queries search tool .

For CPG study retrieval, 2 papers have reported filters that can be applied to MEDLINE. Ingui and Rogers [2] hand searched 4 to 6 selected journals for the years 1991 through 1998 for studies that described the development, validation or evaluation of a CPG, then developed and tested several search filters. Wong *et al *published CPG search strategies for MEDLINE developed by comparing hand searching 161 journals published in the year 2000 with several search terms and applying comparatively, more strict methodological quality criteria [20].

Despite the widespread use of EMBASE, especially in the UK and Europe, little is reported about methodological search filters. Bachmann *et al *[21] and Wilczynski and Haynes [22] reported high performance search strategies for diagnosis studies in EMBASE. In another study, Watson and Richardson tested searches against EMBASE, MEDLINE and PsycInfo for finding randomized controlled trials of cognitive therapy for depression [23]. No studies have yet been published on the retrieval properties of search terms and phrases for CPG studies in EMBASE. To fill this gap in the literature, we applied similar methodology to that used for the identification of CPG study search strategies in MEDLINE [20]. In this report, we describe the information retrieval properties of single terms and combinations of terms for identifying methodologically sound studies of clinical prediction guides in EMBASE.

## Methods

We compared the retrieval performance of methodologic search terms and phrases in EMBASE with a manual review of every article for each issue of 55 journal titles for the year 2000. Originally, 170 journal titles were selected based on recommendations of clinicians and librarians, Science Citation Index Impact Factors provided by the Institute for Scientific Information, and ongoing assessment of their yield of studies and reviews of scientific merit and clinical relevance for the disciplines of internal medicine, general medical practice, mental health, and general nursing practice (list of journals provided by the authors upon request). Of these 170 journals, 135 were indexed in EMBASE. In previous work on search strategy development in MEDLINE, we determined that estimation of search term performance was not substantively affected by using smaller journal subsets, focusing on journals publishing at least some methodologically rigorous articles [unpublished data], and these smaller subsets greatly simplify data processing. Hence, 135 journals were further reduced to a 55 journals that were found to contain at least one study that met our criteria for scientific merit.

When previously developing search strategies for some categories of articles (e.g., therapy, prognosis) for MEDLINE, we split the database into 60% and 40% components to provide a development and validation database. We subsequently found that the comparison between development and validation database results was not statistically significant [24]. For CPG search strategy development for EMBASE, it was not feasible to split the database, as there were too few "pass" articles (e.g., 69 pass CPG articles in EMBASE). Thus, search strategies were developed using the entire database.

Six research staff were rigorously calibrated for hand searching before reviewing the 2000 literature and inter-rater agreement for application of all criteria exceeded 80% beyond chance [25]. Hand searching was performed across 55 journals titles for the year 2000, and methodologic criteria were applied to each item in each issue to determine if the article was methodologically sound for 7 purpose categories (two other types of articles, cost and qualitative studies, were also classified but had no rigor criteria). All purpose category definitions and corresponding methodologic rigor were outlined in a previous paper [25]. Clinical prediction studies were defined as having content that pertains directly to the prediction of some aspect of a disease or condition, and the following methodologic criteria were applied: 1) the guide is generated in one or more sets of real patients (training set); and 2) the guide is validated in another set of real patients (test set).

An initial list of index terms and textwords relating to studies of different purposes (clinical prediction guides, treatment, causation, diagnosis, prognosis, economics, reviews, costs, and studies of a qualitative nature) was compiled in house. The list grew with the addition of terms or phrases suggested by clinicians, librarians and known searchers in the United States and Canada, made upon our request. From here, we compiled a list of 5385 searching terms, of which 4843 were unique and 3524 returned results (terms available on request) for retrieval of studies across all of the purpose categories. Among the 3524 terms were 641 terms that depicted clinical prediction studies such as 'clinical prediction rule', 'derivation set', 'guide', and 'validation cohort', all as textwords; 'validation process', the index term, and the index term 'model', exploded.

The search strategies were treated as "diagnostic tests" for sound studies and the manual review of the literature was treated as the "gold standard." All CPG study search terms and phrases were run in EMBASE and an automated process determined their sensitivity, specificity, precision, and accuracy. Sensitivity for a given topic is defined as the proportion of high quality articles for that topic that are retrieved; specificity is the proportion of low quality articles not retrieved; precision is the proportion of retrieved articles that are of high quality; and accuracy is the proportion of all articles that are correctly classified.

The aim of testing was to identify the best single term, 2-term and multiple-term (greater than two terms) strategies that would optimize sensitivity or specificity or both sensitivity and specificity together. All combinations of terms used the Boolean OR, for example, "predict.tw. OR guide.tw.". (The Boolean AND was not used because this strategy invariably compromised sensitivity.) Next, we tested all 2-term search strategies with sensitivity at least 75% and specificity at least 50% to find multiple term strategies that were optimized for sensitivity. For optimizing accuracy in a multiple term strategy, all 2-term search strategies with accuracy >75% were tested. In total, 36,232 search strategies were tested in the development of clinical prediction guide hedges, which represents the second largest number of strategies (next to cost effectiveness studies) tested among EMBASE strategies investigated within our group.

A logistic regression approach to developing search strategies for MEDLINE did not improve performance [26], so it was not performed for this study.

## Results

Indexing information was downloaded from EMBASE for 27,769 articles of various purpose categories, identified from hand-searching the 55 journals. Of these, 163 (0.58%) were classified as clinical prediction guides, of which 69 (42.3%) were methodologically sound.

Table 1 shows the best single term for high-sensitivity, high-specificity, and best balance of sensitivity and specificity. The single term, predict:.tw., produced both the best sensitivity (78.3%) and best balance of sensitivity (78.3%) and specificity (91.6%). High specificity (98.7%) was achieved using the single term, validat:tw., but with a concomitant drop in sensitivity to 60.9%. Precision ranged from 2.3% to 10.7% for these strategies, reflecting the low prevalence of high quality CPG studies combined with less than perfect specificity.

**Table 1:** Single Term with the Best Sensitivity (keeping Specificity ≥ 50%), Best Specificity (keeping Sensitivity ≥ 50%), and Best Optimization of Sensitivity and Specificity (based on the lowest possible absolute difference between sensitivity and specificity) for Detecting Studies of Clinical Prediction Guides in EMBASE in 2000. Values are percentages (95% confidence intervals).

Search term OVID search*	**Sensitivity (n = 69)**	**Specificity (n = 27700)**	**Precision†**	**Accuracy (n = 27769)**
**Best sensitivity **predict:.tw.	78.3 (68.5 to 88.0)	91.6 (91.2 to 91.9)	2.3 (1.7 to 2.9)	91.5 (91.2 to 91.7)
**Best specificity **validat:.tw.	60.9 (49.4 to 72.4)	98.7 (98.6 to 98.9)	10.7 (7.6 to 13.7)	98.6 (98.5 to 98.8)
**Best Optimization of Sensitivity & Specificity **predict:.tw.	78.3 (68.5 to 88.0)	91.6 (91.2 to 91.9)	2.3 (1.7 to 2.9)	91.5 (91.2 to 91.7)

*The search strategy is reported using Ovid's search engine syntax for EMBASE.

†Denominator varies by row. : = truncation; tw = textword (word or phrase appears in title or abstract).

Combination of terms with the best results for sensitivity, specificity and optimization of sensitivity and specificity are shown in Table 2. When multiple terms are run, nearly all measures for sensitivity and specificity improve over results for single term strategies. The 3-term strategy predict:.tw. OR exp methodology OR validat:.tw achieved the best sensitivity (97.1%), with a specificity of 74.2%. Specificity peaked at 98.8%, as did precision (10.8%) and accuracy (98.7%), with the 2-term search phrase validation.tw. OR prediction.tw., but with a significant drop in sensitivity to 60.9%. The strategy that performed best for optimization of sensitivity and specificity was validat:.mp. OR index.tw. OR model.tw, measured at 91.3% and 90.2%, respectively. This 3-term strategy outperformed the best single term search strategy by 13% in sensitivity, while maintaining a comparable value for specificity.

**Table 2:** Combination of Terms with the Best Sensitivity (keeping Specificity ≥ 50%), Best Specificity (keeping Sensitivity ≥ 50%), and Best Optimization of Sensitivity and Specificity (based on abs [sensitivity-specificity]<1%) for Detecting Studies of Clinical Prediction Guides in EMBASE in 2000. Values are percentages (95% confidence intervals).

Search Strategy OVID search*	**Sensitivity (n = 69)**	**Specificity (n = 27700)**	**Precision†**	**Accuracy (n = 27769)**
**Best Sensitivity **predict:.tw. OR exp methodology OR validat:.tw.	97.1 (93.1 to 100.0)	74.2 (73.7 to 74.7)	0.9 (0.7 to 1.2)	74.2 (73.7 to 74.7)
**Best Specificity **validation.tw. OR prediction.tw.	60.9 (49.4 to 72.4)	98.8 (98.6 to 98.9)	10.8 (7.7 to 13.9)	98.7 (98.5 to 98.8)
**Best Optimization of Sensitivity & Specificity **validat:.mp. OR index.tw. OR model.tw.	91.3 (84.7 to 98.0)	90.2 (89.9 to 90.6)	2.3 (1.7 to 2.8)	90.2 (89.9 to 90.6)

*Search strategies are reported using Ovid's search engine syntax for EMBASE.

†Denominator varies by row. : = truncation; tw = textword (word or phrase appears in title or abstract); exp = exploded subject heading; mp = multiple posting – term appears in title, abstract, or subject heading.

## Discussion

In this study we report search filters found to be effective for the retrieval of clinical predication guide studies from EMBASE. These filters are optimized for sensitivity, specificity or best sensitivity and specificity combined, each lending the searcher unique results that can be geared to his/her needs. The strategy optimized for sensitivity should be applied in cases where retrieval of all relevant articles is key, and substantial weeding of irrelevant content is seen to be acceptable. The most specific search filter is effective when the aim of the search is to retrieve only highly relevant articles, where inclusion of all pertinent matter is less important. Where the intention is to uncover a balance of targeted hits with off topic material then the strategy that maximizes both sensitivity and specificity would be best.

When comparing the results of this study to that reported by Wong et al [20] for CPGs in MEDLINE, several similarities can be drawn. Both studies report low precision for most search strategies. Like the MEDLINE search strategies, low precision is attributed to the varying content of the EMBASE database, a small proportion of which are studies of clinical prediction guides. Precision may be improved with the application of the "AND" / "AND NOT" Boolean operators or the addition of clinical content terms or journal subsets using the Boolean AND along with the methodology search filters, but these tactics are likely to compromise sensitivity.

Other parallels occur between the 2 reports with respect to best identified search strategies. For single terms, predict (as a textword in EMBASE, predict:.tw., and as a "multiple posting" term in MEDLINE, predict:.mp.) achieved both highest sensitivity and best optimization for sensitivity and specificity in both EMBASE and MEDLINE. Similarly, validat:.tw. generated best results for specificity in both databases.

It is interesting to note that no indexing terms contributed to the optimized search strategies for CPG studies in EMBASE or MEDLINE; textwords were the composite for all winning strategies. This finding is consistent with indexing terminology not keeping pace with research methods, and suggests a means for improving indexing and retrieval in the future.

For practical purposes, we restricted our methods filter to just 2 criteria, the use of both a training and test set. Additional criteria that could have been applied include prospective validation, stating the mathematical technique used to develop the rule, clear definition and blinded assessment of predictor variables and outcomes, and prospectively testing the effect of the rule in clinical practice [1]. Further research would be needed to determine the performance of our search strategies for these additional criteria. However, it is predictable that adding more criteria would diminish the yield of our search strategies. For example, Laupacis et al [1] found that only 3% of studies of CPGs prospectively tested clinical use, whereas we found that 42% of CPG articles passed our filter. Thus, rather than incorporating additional criteria into the derivation of search strategies, we would suggest that the additional criteria be applied by end-users, to articles retrieved by our strategies, if relevant to their purposes.

For clinicians to be able to make optimal use of clinical prediction guides in their practice, their accessibility needs to be improved upon. This study highlights search terms that maximize the retrieval of CPG studies, as well as illustrating that there is room for improvement, especially in precision. The application of "AND" and "AND/NOT" combinations or multivariate statistical techniques may help, but this remains to be determined.

## Conclusion

The retrieval of higher quality clinical prediction guides from EMBASE is facilitated by the application of search filters that have been optimized for sensitivity or specificity or both.

## List of Abbreviations

Clinical Prediction Guide: CPG

## Competing Interests

The author(s) declare that they have no competing interests.

## Authors' Contributions

NLW and RBH prepared grant submissions in relation to this project. JH, NLW and RBH drafted, commented on and approved the final manuscript. NLW and RBH supplied intellectual content to the collection and analysis of the data. NLW participated in the data collection and all authors were involved in data analysis.

## Pre-publication history

The pre-publication history for this paper can be accessed here:


